# Recurrent nasopharyngeal rhinosporidiosis: Case report from Qatar and review of the literature

**DOI:** 10.1016/j.idcr.2020.e00901

**Published:** 2020-07-03

**Authors:** Gawahir M. Ali, Wael Goravey, Samir A. Al Hyassat, Mahir Petkar, Muna A. Al Maslamani, Hamad Abdel Hadi

**Affiliations:** a Department of Infectious Diseases, Communicable Diseases Centre, Qatar; b Department of Laboratory Medicine and Pathology, Hamad Medical Corporation, Qatar

**Keywords:** Rhinosporidiosis, Mucosal polyps, Dapsone, Qatar

## Abstract

Rhinosporidiosis is a chronic granulomatous infectious disease that predominantly affects the mucosal membranes of the nose and nasopharynx. The disease is caused by Rhinosporidium seeberi, an eukaryotic pathogen with distinct geographical distribution particularly in tropical and subtropical areas acquired mainly through aquatic exposure.

We report a case of a young Nepalese male who presented with recurrent right nasopharyngeal mass where surgical excision and histopathological examination confirmed the diagnosis following distinct pathognomonic findings. There is no optimal effective management of the disease and surgical excision coupled with cauterization to prevent recurrence is the recommended best option since medical treatment alone is ineffective. The clinical presentation, assessment and management options are reviewed.

## Highlights

• Exotic tropical infection in migrant populations.
• Intriguing history.
• Enigmatic taxonomy.
• Challenging management.

## Introduction

Rhinosporidiosis is a chronic granulomatous disease caused by Rhinosporidium seeberi which has a confusing historic taxonomy spanning fungi, parasite and bacteria till eventually classified as an aquatic eukaryote, based on comparison with similar aquatic organisms and genetic sequencing [1,2]. Although the disease has been reported worldwide, it is mainly endemic in tropical and subtropical countries. Aquatic exposure is almost universal in all cases and transmission is mainly through direct contact with stagnant water [3]. Nasopharyngeal Rhinosporidiosis chiefly presents with polypoid tumor like masses in affected mucosal sites. Because of the friable and pendulous nature of these lesions, presenting complaints are mainly of nasal symptoms of rhinorrhea, epistaxis or ultimately obstruction. Assessment mainly relies upon the clinical presentation of the characteristic lesions together with typical appearances upon histological examination [4].

## Case description

**Fig. 1:**
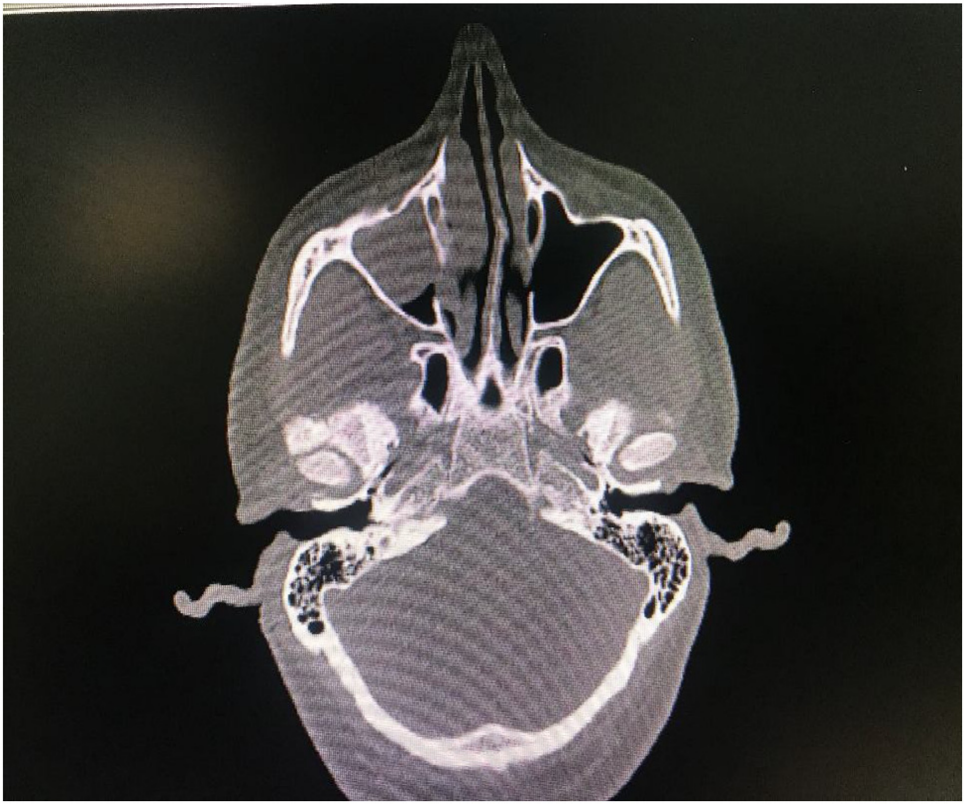
Axial CT head showing: almost total opacification of the right maxillary sinus with concentric soft tissues mass protruding into the nasal cavity deviating the nasal septum.

A 35-year-old Nepalese male presented acutely to our medical service with right sided epistaxis of two-day duration. The patient had no chronic medical conditions and previously was working as a farmer in his home country. Further evaluation revealed previous similar episodes in the preceding year, with history of excision of hard masses in the past. Upon clinical assessment, a right-sided polypoid strawberry nasopharyngeal mass was seen. Blood investigations were unremarkable. Computerized tomography of the nose and sinuses showed a lobulated soft tissue mass occupying the right nasal cavity near the middle and inferior nasal turbinates protruding into the right nasopharynx (Fig. 1).

**Fig. 2:**
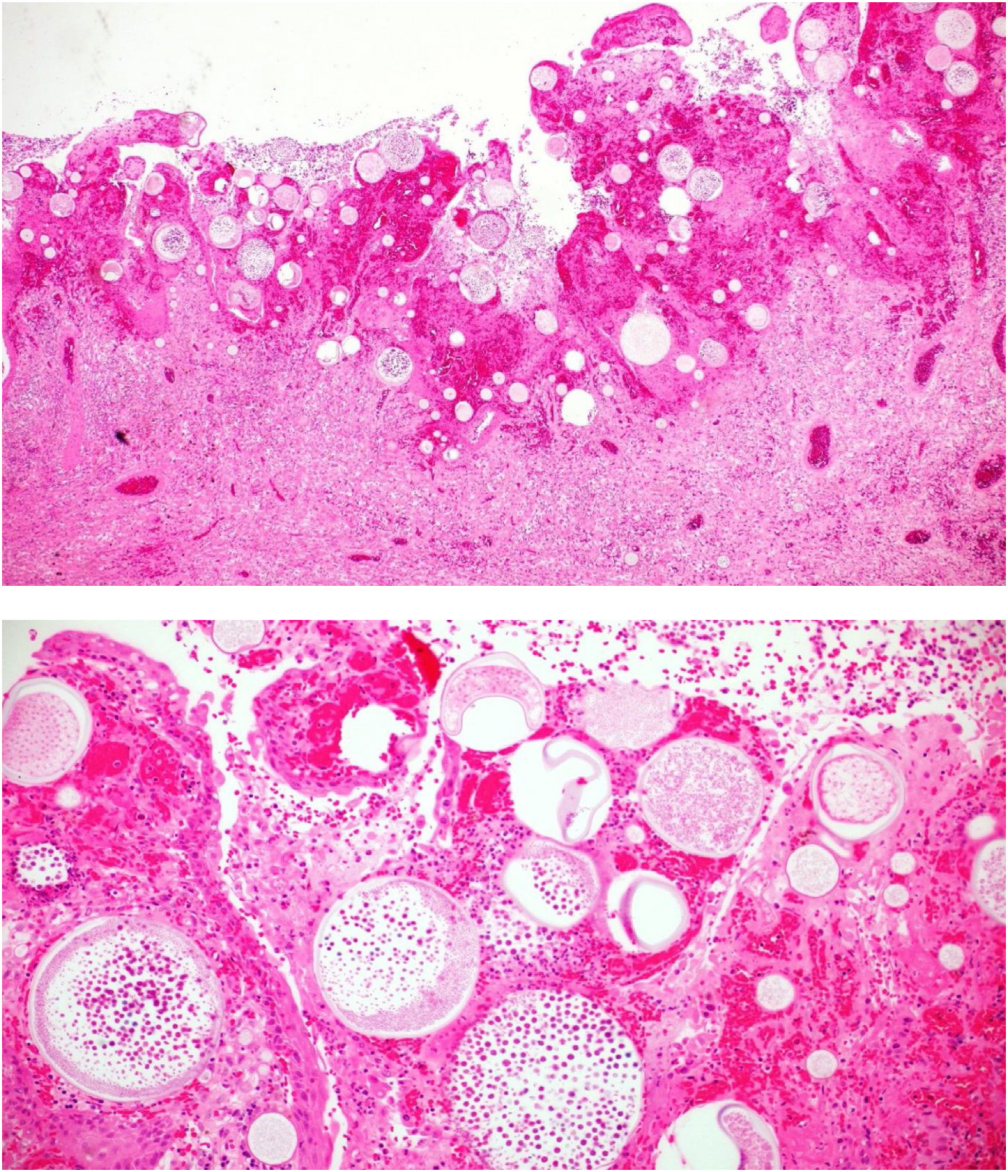
H & E staining: A- Low power magnification showing characteristic thick walled sporangia in various stages of development, containing endospores. B- High power view with numerous endospores noted within the sporangia.

Following initial evaluation, the bleeding was halted followed by functional endoscopic sinus procedure for excisional surgery. Intraoperative findings were of a large polypoid friable mass filling the right nasal cavity attached to the inferior and middle turbinates which bled easily. Complete excision of the mass together with partial inferior turbinectomy was performed followed by cauterization. Subsequent histopathological examination demonstrated typical spores within the characteristic pathognomonic sporangia, confirming the diagnosis of naso-sinal Rhinosporidiosis (Fig. 2). On detailed past medical history evaluation, it transpired the patient had been previously treated for a similar condition with partial excisional polypectomy followed by a one-year Dapsone therapy. Despite these measures, the patient relapsed with the current recurrence.

The diagnosis was made based on the patient clinical profile, radiological assessment, intra-operative findings together with the characteristic histopathological results. Reviewing the evidence from medical literature we found no strong supporting evidence for repeating medical treatment alone with associated recurrence rates. Complete surgical excision with adjunct cauterization was the best recommended treatment option, one year into follow up there is no further recurrence.

## Discussion

Rhinosporidiosis is a rare enigmatic infectious disease with a historic contentious etiology which was only revealed over recent decades. The disease is caused by Rhinosporidium seeberi, a hydrophilic organism that was initially oscillating in classification between parasites, fungi as well as bacteria till it was revealed as an aquatic eukaryotes that infects human and animals, towards the end of the 20st century [3]. The name was coined after R.Seeber who first described the clinical presentation in Argentina and proposed a probable pathological etiology at the beginning of the 20th century [5].

Rhinosporidiosis primarily affects aquatic animals like fish and reptiles while humans as well as large domestic mammals and birds are incidental hosts [6]. The disease is not uncommon in tropical and subtropical areas with preponderance clustering in major endemic regions around the Indian subcontinent particularly south India and Sri Lanka together with historic endemic areas in South America [3]. Most affected patients are usually adults below the age of 40 with male to female ratio of about 2.5:1 [4]. Traditionally transmission has been linked to contact with stagnant lakes and ponds, with occupations linked to these activities where frequent exposure occurs [7]. Other modes of transmission like autoinfection, hematogenous and lymphatic spread have also been reported [2].

Rhinosporidiosis has a predilection for external mucosal tissues like the nasopharynx and around the eyes, probably because of vulnerability together with probable need for wet surfaces for pathogen attachment and proliferation [1]. The disease can affect any mucosal sites in the human body presenting usually as tumor-like masses. The most affected sites in term of frequency are the mucosal surfaces of nasopharynx followed by ocular, cutaneous, genital and respiratory mucosa [2,8]. Naso-pharyngeal Rhinosporidiosis usually has an insidious onset. The vascular friable polyps with white projections, which can easily bleed give it the description of “strawberry” appearance. Patient can be asymptomatic or characteristically describe foreign body sensation, rhinorrhea, nasal obstruction and epistaxis [9]. All recurrent presentations of our patient were with epistaxis and the clinical assessment described the nasal lesion as strawberry mass, which easily bled when handled.

Recurrence is a well-known feature of Rhinosporidiosis even after complete excision which might be explained by a decrease in anti-rhinosporidial cell-mediated immunity mediated by switch from Th-1 to Th-2 [2].Our patient is a native of Rhinosporidiosis endemic region in southeast Asia giving an obvious example of this phenomenon with the third recurrence in a decade. During our assessment, we managed to obtain a history of frequent activities related to exposure to stagnant water to establish the epidemiological link. It is important to recognize tropical infections in immigrants since it has been observed that socioeconomic migration was the main reason of diagnosis of exotic tropical diseases in non-endemic areas [10].

For practical reasons, it has been advised to consider Rhinosporidiosis in any patient coming from endemic regions with nasal masses [3]. Radiological investigation particularly CT and MRI imaging are extremely helpful in outlining the extent of the disease as well as excluding other pathology in patients presenting with nasopharyngeal masses [3]. The diagnosis can be established through advanced molecular techniques like PCR or 16 s RNA or simply by findings the characteristic mucosal appearances upon histopathological examination [2,3].

The recommended management for Rhinosporidiosis particularly nasopharyngeal presentation is wide surgical excision with cauterization of the base of the lesion to prevent recurrence [3,11]. Despite these measures; recurrence rates remain high probably due to spillage and seeding of sporangia during removal, that invade adjacent normal tissues [12]. Currently; there is not enough supporting medical evidence to support alternative medical therapy alone. Prolonged treatment with Dapsone or antifungals has been tried in the past with variable success, in selected cases but with very high rates of recurrence [12,13]. The failure of medical therapy is attributed to impenetrability of the sporangial wall to most antimicrobial agents, and most importantly, difficulties in culturing the organism making susceptibility testing futile [2].

## Conclusion

Otolaryngologists and infectious diseases physicians should keep in mind the presentation of Rhinosporidiosis when managing migrant population with nasopharyngeal masses or abnormal growths related to mucosal surfaces even from non-endemic areas. Assessment with radiological imaging together with the characteristic histological findings are crucial steps in management. Since there is no clear proven benefit of medical therapy, the recommended treatment for Rhinosporidiosis particularly the commonest nasopharyngeal presentation, is wide surgical excision together with cauterization of the excised sites.

## Consent compliance with ethical standards

A written informed consent was obtained from the patient to include clinical presentation together with results and imaging. This was subsequently reviewed and approved by the institution ethics and research review board.

## Funding

No funding was received towards the publication

## Declaration of Competing Interest

Authors declared no competing interests in relation to current publication.
